# Effects of Articular Cartilage Constituents on Phosphotungstic Acid Enhanced Micro-Computed Tomography

**DOI:** 10.1371/journal.pone.0171075

**Published:** 2017-01-30

**Authors:** Sakari S. Karhula, Mikko A. Finnilä, Mikko J. Lammi, Janne H. Ylärinne, Sami Kauppinen, Lassi Rieppo, Kenneth P. H. Pritzker, Heikki J. Nieminen, Simo Saarakkala

**Affiliations:** 1 Research Unit of Medical Imaging, Physics and Technology, Faculty of Medicine, University of Oulu, Oulu, Finland; 2 Infotech Doctoral Program, University of Oulu, Oulu, Finland; 3 Department of Applied Physics, University of Eastern Finland, Kuopio, Finland; 4 Medical Research Center Oulu, Oulu University Hospital and University of Oulu, Oulu, Finland; 5 Department of Integrative Medical Biology, University of Umeå, Umeå, Sweden; 6 School of Public Health, Health Science Center of Xi’an Jiaotong University, Key Laboratory of Trace Elements and Endemic Diseases, National Health and Family Planning Commission, Xi’an, P. R. China; 7 Department of Laboratory Medicine and Pathobiology, University of Toronto, Toronto, Canada; 8 Mount Sinai Hospital, Toronto, Canada; 9 Department of Physics, University of Helsinki, Helsinki, Finland; 10 Department of Diagnostic Radiology, Oulu University Hospital, Oulu, Finland; Drexel University, UNITED STATES

## Abstract

Contrast-enhanced micro-computed tomography (CEμCT) with phosphotungstic acid (PTA) has shown potential for detecting collagen distribution of articular cartilage. However, the selectivity of the PTA staining to articular cartilage constituents remains to be elucidated. The aim of this study was to investigate the dependence of PTA for the collagen content in bovine articular cartilage. Adjacent bovine articular cartilage samples were treated with chondroitinase ABC and collagenase to degrade the proteoglycan and the collagen constituents in articular cartilage, respectively. Enzymatically degraded samples were compared to the untreated samples using CEμCT and reference methods, such as Fourier-transform infrared imaging. Decrease in the X-ray attenuation of PTA in articular cartilage and collagen content was observed in cartilage depth of 0–13% and deeper in tissue after collagen degradation. Increase in the X-ray attenuation of PTA was observed in the cartilage depth of 13–39% after proteoglycan degradation. The X-ray attenuation of PTA-labelled articular cartilage in CEμCT is associated mainly with collagen content but the proteoglycans have a minor effect on the X-ray attenuation of the PTA-labelled articular cartilage. In conclusion, the PTA labeling provides a feasible CEμCT method for 3D characterization of articular cartilage.

## Introduction

Composition of articular cartilage (AC) is known to be strongly associated with its biomechanical properties [1]. Main constituents in AC are proteoglycans (PG), collagens and chondrocytes. Osteoarthritis (OA) progression leads to loss of PG content, degeneration of collagen network with perichondronal collagen condensation, and focal intra matrix collagen formation, all resulting in a progressive heterogeneity of cartilage matrix and a deterioration of AC biomechanical properties [2]. Therefore, investigation of the histologic distribution and microarchitectural changes of these macromolecular constituents is important for understanding OA progression.

Contrast-enhanced micro-computed tomography (CEμCT) enables high-resolution 3D characterization of soft tissues [3] including AC [4]. Several studies have shown the potential of CEμCT to quantify the PG content and distribution in the AC both *in vitro* and *in vivo* [5–10]. These studies utilize either negatively charged (Hexabrix®, Magnevist®) or positively charged (CA1+, CA2+, CA4+ and Tantalum Oxide Nanoparticles) contrast agents to produce contrast in the AC. Hexabrix® and Magnevist® are also in clinical use to detect the loss of PGs [11,12]. However, it can be debated whether the decrease in the PG content is primarily followed by changes in the collagen integrity. For example, the swelling of the AC resulting from the collagen degradation, can lead to ostensible PG loss [13,14]. Consequently, novel methods, which are able to specifically quantify the constituents of the AC in the micro-level, are needed to understand the disease progression and improve the diagnostics of OA.

In our previous study, we reported the potential of CEμCT method for analyzing the 3D collagen distribution in AC by using phosphotungstic acid (PTA) as the contrast agent [4]. The low pH of the PTA solution (2.71) enables the binding of the negatively charged PTA to the collagen, which has a positive net charge in low pH [15]. Since PGs have a small negative charge in the same pH range[15], it is reasonable to assume that the PGs may restrict the PTA binding to the collagen in the AC by means of electrostatic repulsion. Furthermore, the actual specificity of the PTA labeling used in CEμCT for the collagen in the presence and absence of PGs in the AC is not known. Previously, it has been shown that enzymatic digestion of PGs increases Hexabrix® (ioxaglic acid) diffusion into the cartilage, thus, increasing the cartilage X-ray attenuation in the CECT imaging [16,17]. Similarly to the PTA, the ioxaglic acid is also negatively charged, and its ability to reveal the PG distribution in AC relies on the repulsion by the charge of PGs. Ioxaglic acid also differs from the PTA by the weaker negative charge. Because the Hexabrix® is tailored for clinical applications, it is dissolved in a solution, which has pH of approximately 7.4, where the net charge of the collagen is close to zero [15]. Therefore, there is likely no electrochemical attraction between the negatively-charged ioxaglic acid and the collagen network of AC.

The aim of this study was to investigate the specificity of the PTA for the collagen content and distribution in bovine AC in the CEμCT setting by enzymatically degrading either the collagen or the PGs. The enzymatic degradations were validated using Fourier-transform infrared imaging (FTIRI), histology and biochemical analyses for the contents of the collagen and the PGs [18,19]. Furthermore, PTA distribution in AC was compared with Hexabrix® distribution in CEμCT settings. Our hypothesis was that the binding of the PTA is strongly affected by collagen degradation, while the degradation of the PGs has only a minor effect on the PTA binding.

## Materials and Methods

### Sample Preparation

**Fig 1:**
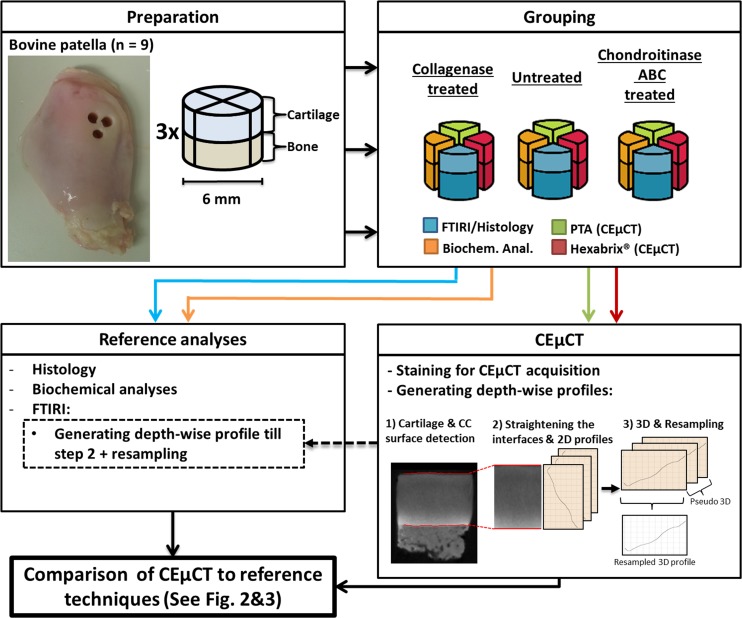
Preparation, grouping and analysis of the samples. Sample procedure is shown in the flow chart. Cylindrical (diameter = 6 mm) osteochondral samples (n = 3) were collected from visually healthy patella of nine bovines, sectioned in pieces and divided into groups depending on their enzymatic treatment. Pieces from each group were subjected to 1) contrast-enhanced computed micro-tomography (CEμCT) with phosphotungstic acid (PTA), 2) CEμCT with Hexabrix®, 3) Fourier-transform infrared imaging (FTIRI) and histology, and 4) biochemical analyses. The generation of the depth-wise profiles from CEμCT and FTIRI data consisted of three steps: 1) cartilage surface and the uncalcified cartilage-calcified cartilage (CC) interface detection, 2) Straightening the interfaces and generation of 2D profiles for each image, 3) generation of the 3D profile by averaging corresponding depth-wise coordinates throughout slices and resampling the depth-wise profile to 100 datapoints. FTIRI data was only resampled to 100 datapoints in the third step. For more specific description of this process, see the Methods section. The CEμCT results were compared with the reference analyses (FTIRI, histology and biochemical analyses).

Right hind limbs from bovines (*N* = 9) were acquired from the local abattoir (Veljekset Rönkä Oy, Kemi, Finland) and stored in a freezer (-20°C) until experiments. After thawing, three adjacent cylindrical osteochondral plugs (diameter = 4.8 mm) were drilled from the lateral upper quadrant of each patella and divided into (i) collagenase, (ii) chondroitinase ABC and (iii) control (untreated) groups, respectively (Fig 1). Before enzymatic treatments, each plug was cut into four pieces. The first and the second pieces were subjected to CEμCT using the PTA and the Hexabrix® as contrast agents, respectively. The third and fourth pieces were used for reference techniques, which included histology, Fourier Transform Infrared Imaging (FTIRI) and biochemistry.

### Enzymatic degradations

Chondroitinase ABC (Sigma-Aldrich, St. Louis, MO, C3667; 0.1 U/ml, 44 h at 37°C, 5% CO_2_) in phosphate-buffered saline (PBS, pH 7.4) and collagenase (Sigma-Aldrich, C0773; 30 U/ml, 50 h at 37°C, 5% CO_2_) in PBS (pH 7.4) were used to degrade chondroitin/dermatan sulfate and hyaluronan glycosaminoglycans (GAGs), and the collagens, respectively [20]. The control samples were directly subjected to CEμCT, histology, FTIRI and biochemical analyses.

### CEμCT

After enzymatic treatments, samples for the PTA labeling were first fixed in 10% neutral-buffered formalin for 5 days and transferred to 70% ethanol for 8 hours followed by 36 h immersion in the 1% w/v PTA solution (Sigma-Aldrich, 79690) in 70% ethanol. The PTA labelling protocol was same for the adjacent control samples. The PTA labelled samples were wrapped in cellulose and immersed in a sealed container filled with 70% ethanol. The other set of the CEμCT samples were immersed after the enzymatic treatment in 10% Hexabrix® 320 (Mallinckrodt Pharmaceuticals Inc., St. Louis, MO, USA) in PBS for 28 h and subjected to μCT imaging inside a sealed container. Control samples for the CEμCT with Hexabrix® were subjected to the same protocol immediately after the core extraction. Due to the relatively fast washout of the Hexabrix®, the container could not be filled with liquid. To prevent the drying of the sample during the scan, moistened cellulose was added on the bottom of the container to raise the humidity inside the container.

The CEμCT scanning was conducted with the Skyscan 1176 (Bruker microCT, Kontich, Belgium; settings: 40 kV, 600 μA, 1200 projections, exposure 200 ms/frame, average of 2 frames per projection, isotropic voxel side length 17.4 μm, without additional filtration). Reconstructions for X-ray projections were made with the manufacturer’s Skyscan Nrecon-software (v. 1.6.9, Bruker microCT). Ring artefact and beam hardening corrections were applied in the reconstruction.

For statistical analyses, depth-wise profiles were calculated from the reconstructed CEμCT image stacks (Fig 1). Depth-wise profiles were generated from 3D datasets with Matlab R2014a software (v. 8.3.0.532, Mathworks, Natick, MA, United States) (Fig 1). Prior to generating the depth-wise profiles, 3D image stacks were rotated so that cartilage surface and uncalcified cartilage-CC interface were oriented horizontally in the image stack. To prevent possible artifacts on the edges of the plugs (from the preparation and enzymatic treatment) affecting the depth-wise analysis, z-direction was restricted to 20 voxels (20 slices from reconstructed stack, see Fig 1). Datasets were processed slice-by-slice (pseudo-3D), and the generation of the depth-wise profiles consisted of three steps: cartilage surface and uncalcified cartilage-CC interface detection, depth-wise vector normalization and 2D profile generation, and 3D profile generation and resampling. Interface detections were done by first applying a global threshold to binarize the image and then detecting the depth-wise co-ordinates for the interface in each image column. Two separate global thresholds were used for cartilage surface and uncalcified cartilage-CC interface. This simplified interface detection was possible because of the different Hounsfield unit (HU) values for calcified tissue (CC & bone) and PTA-stained cartilage in μCT imaging. In the second step for generating the depth-wise profiles, the interfaces were straightened as described in a previous study [4]. After straigthening the interfaces, the image rows were averaged and 2D profiles for each slice was generated. In the third step, the 2D profiles were averaged by corresponding depth-wise co-ordinates throughout slices to generate the 3D depth-wise profiles. These 3D depth-wise profiles were finally resampled to 100 datapoints.

For the enzymatic treated samples each datapoint (x_Enz_) was compared to the corresponding datapoint in the control group (x_con_). The comparison was done by calculating the relative change (see Eq 1) sample-by-sample and calculating the depth-wise means and standard deviations for both comparison groups (collagenase vs. control, chondroitinase ABC vs. control).

$$Relative\;difference\;(\%)=100*\frac{x_{Enz}-x_{con}}{x_{con}}$$ (1)

### Histological analyses

To qualitatively evaluate the effectiveness of enzymatic degradations, 3 μm sections were prepared for Picrosirius red and Safranin O [21] stains to determine the collagen and the GAG distributions, respectively. The Picrosirius Red -staining protocol used in our study was modified from the standard protocol [22] by adding the purification step to demonstrate collagen fibre patterns. This was conducted by 18 h immersion in 0.5% papain (Acros Organics, 416761000) in 0.05 M PBS (pH 4.4, 37°C) and 1000 U/ml hyaluronidase (Sigma-Aldrich, H3506) in 0.1 M PBS (pH 6.9, 37°C) digestions before the staining with 0.1% Direct red 80 in saturated picric acid (Sigma-Aldrich, 365548). Polarized light microscopy (PLM) was used to measure the retardance caused by picrosirius red stain in the sections to reveal alterations in the collagen content. The sections were imaged with Abrio polarized light imaging system (CRi, Inc., Woburn, MA, USA) mounted on a light microscope (Nikon Diaphot TMD, Nikon, Inc., Shinagawa, Tokyo, Japan).

### FTIRI analyses

The unstained microscopic sections (thickness = 5 μm) were imaged with Hyperion 3000 FTIRI Microscope (Bruker Inc., Billerica, MA, USA) with the following parameters: binning of 2 x 2, pixel size of 5.4 x 5.4 μm, spectral resolution of 8 cm^-1^, and 16 acquisitions per pixel. The acquired spectral data were offset-normalized, and the integrated absorbances of the amide I region (1595–1720 cm^-1^) and the carbohydrate region (985–1120 cm^-1^) were calculated to indicate the distribution of the collagen and the GAGs, respectively [19,23]. Depth-wise profiles for FTIRI were generated similarly as in CEμCT analysis with slight modification as after generating the 2D profile from single image, the profile was resampled to 100 datapoints without further processing. The relative differences were calculated from the profiles similarly as for the CEμCT profiles (see. Eq 1).

### Uronic acid quantification

The pre-weighted samples were digested with papain (1 mg/ml in 5 mM cysteine and 5 mM EDTA) overnight at 60°C to solubilize the cartilage tissue. Uronic acid content was then determined [24], and the results were normalized by wet weight.

### Proteoglycan structure analysis

The cartilage sections reserved for biochemical analyses were cut into smaller pieces, and the PGs were extracted by immersing the samples in 4 M guanidinium hydrochloride in 50 mM sodium acetate, 10 mM EDTA and 5 mM benzamidine hydrochloride (pH 5.8) at 4°C for 72 hours. The amount of the PGs was determined with dimethylmethylene blue assay [25], and the PGs (5 μg GAG) were separated in 1.2% agarose gels as described previously [26].

### Statistical analyses

Depth-wise profiles from CEμCT and FTIRI were evaluated point-by-point with the 2-tailed Wilcoxon signed-rank test (IBM SPSS Statistics software v. 21, International Business Machines Corporation, Armonk, NY, U.S) by comparing the means of enzymatic degradation groups with the means of control group. The differences in the agarose gel profiles between the control and the enzymatically treated ones were tested with the 2-tailed paired t-test.

To estimate the normal biological variation within the samples, coefficients of variation (CV, ratio between the standard deviation and the mean) in the pixel/voxel values from the control (untreated) samples through the tissue depth were calculated.

## Results

**Fig 2:**
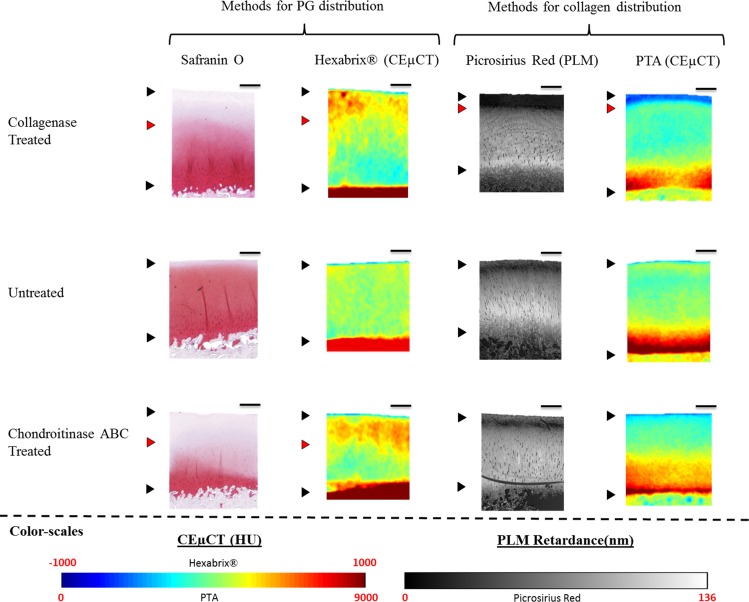
2D-slices of PTA- and Hexabrix®-labelled ACs (CEμCT) compared to the corresponding histological collagen and PG stains. Birefringence of Picrosirius red stain was imaged with polarized light microscopy (PLM) to visualize the collagen fibre content and distribution. The histological stains showed clear effects of the enzymes, which can be seen as an insufficient staining in the surface of the sample as compared to the control samples (the depth of the enzyme effect indicated with red arrows). The Picrosirius red stain and the PTA showed highly similar collagen distributions. Also Safranin O and Hexabrix® showed highly similar PG distributions. Color-scaled Hounsfield unit (HU) -values of the CEμCT and retardance (nm) of PLM are presented in the bottom. Different scalings between the PTA- and Hexabrix®-enhanced images were used to better visualize the distributions of the contrast agents in the cartilage. Black arrows indicate the cartilage surface and the uncalcified cartilage-CC interface. Scale bars on the right corner of each sample equal to 500 μm.

The visual comparison between the CEμCT images of the PTA-labelled AC and histological staining for the collagen distribution in the AC (Picrosirius Red with PLM) was consistent (Fig 2). As expected, the visual comparison between the histological PG staining (Safranin O) and the X-ray attenuation of PTA in AC did not correspond to each other. Comparison between the Safranin O staining and the X-ray attenuation obtained from CEμCT of AC with PG-sensitive Hexabrix® revealed the close correspondence between the ioxaglic acid distribution and the PG distribution. The X-ray attenuation from CEμCT with Hexabrix® did not have correlation with the Picrosirius red staining.

**Fig 3:**
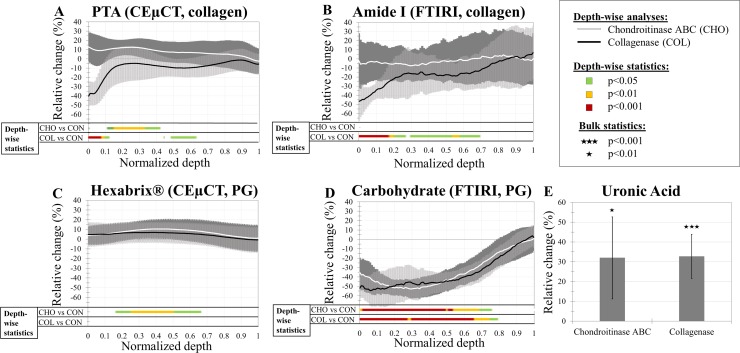
The relative changes between the enzymatic treatment groups and the control (untreated) group. For depth-wise analyses (A-D), the tissue depth was normalized, depth-wise profiles were calculcated and resampled to 100 points from CEμCT (in 3D) and FTIRI (in 2D). The relative change and statistical differences were calculated point-by-point for both enzymatic treatments (see S1 Table for p-values throughout depth). Statistically significant difference between enzymatically treated groups and control group was calculated with Wilcoxon signed-rank test. After collagenase treatment, decreasing relative change in the X-ray attenuation obtained from PTA-stained AC (A) was significant in the surface and deeper layers of the tissue. Similar decrease with the reference collagen content measured with FTIRI (amide I) (B) was observed in the same areas. After chondroitinase treatments, both CEμCT techniques (A and C) had significant decrease in the X-ray attenuation in the middle layers of tissue (PTA: 13–39% of the tissue depth; Hexabrix®: 22–48% of the tissue depth). Reference PG content measured with FTIRI (D) and uronic acid analysis (E) both indicated a significant decrease of PG content with both enzymatic degradations.

A statistically significant decrease of 14–40% in the X-ray attenuation of PTA was observed in the surface (*p*<0.05 in range of tissue depth 0–13%, see S1 Table for specific p-values throughout the tissue) of the collagenase-degraded samples compared to the control group (Fig 3). Smaller (9–10%), but a statistically significant decrease in the X-ray attenuation was observed deeper in the tissue (tissue depth 48–63%). These results from μCT of PTA-stained AC corresponded spatially with the collagen loss observed in the collagenase-digested specimens by the FTIRI (amide I absorbance) analyses (Fig 3). Furthermore, the FTIRI analyses, based on the carbohydrate region (Fig 3), showed a significant (*p*<0.05) decrease in the PGs in the tissue depth of 0–77%. However, there was no statistically significant increase in X-ray attenuation of Hexabrix® in CEμCT (Fig 3).

After chondroitinase ABC degradation, statistically significant changes (*p*<0.05 in the tissue depth of 13–39%) were also observed in the X-ray attenuation of PTA in AC even though the FTIRI-based absorbance from the amide I peak (Fig 3) did not show statistically significant changes between the groups. However, a significant decrease (*p*<0.05) in the FTIRI-based absorbance for the carbohydrate region after PG degradation in the tissue depth 0–73% (Fig 3), and a significant increase (*p*<0.05) in Hexabrix®-enhanced μCT attenuation was observed in the tissue depth 22–48% (Fig 3). The uronic acid analysis confirmed the significant (chondroitinase ABC: *p* = 0.001, collagenase: *p* = 0.019) loss of GAGs in both enzyme digestions (Fig 3).

**Fig 4:**
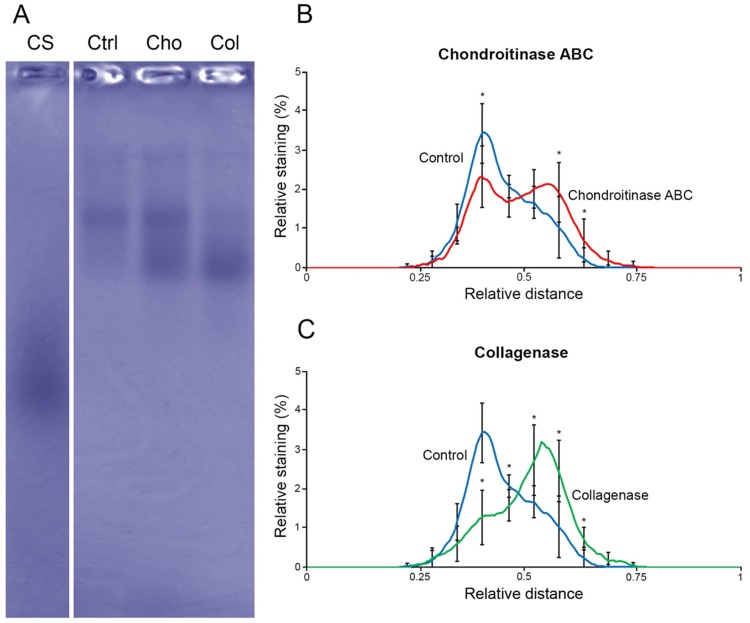
Structural analysis of PGs by agarose gel electrophoresis. Representative lanes of chondroitin sulfate (CS), and PGs extracted from the control (Ctrl), chondroitinase ABC-treated (Cho) and collagenase-treated (Col) samples are shown (A). The mean profiles of the samples for control vs. chondroitinase ABC-treated (B) and control vs. collagenase-treated (C) samples are shown. The migration distance of CS was set to reference value 1.00. The paired t-test was used to estimate the difference in the staining profiles. Statistically significant differences (*) for chondroitinase ABC from left to right were *p* = 0.0042, *p* = 0.0032, *p* = 0.0220; and for collagenase from left to right were *p* = 0.0001, *p* = 0.0315, *p* = 0.0220, *p* = 0.0308, *p*<0.001. Only a limited set of standard deviations are shown for the clarity of the figure (n = 8).

Since the chondroitinase ABC and collagenase treatments led to practically similar loss of GAGs from the tissue, we analysed the structures of the major PGs with agarose gel electrophoresis. The analysis showed a marked increase in the presence of diffuse higher mobility PGs (Fig 4A), indicating a partial degradation of the large PGs (apparently aggrecans) by both enzyme treatments. The average profiles of chondroitinase ABC (Fig 4B) and collagenase-treated (Fig 4C) samples revealed significant differences in the staining patterns.

Additionally, the coefficient of variation (CV) in untreated group was calculated to provide information about the biological variation between the samples. Biological sample-to-sample variations in the X-ray attenuation of the PTA-labelled AC varied in range ~8–16% at different tissue depths (untreated samples). Biological variations of Hexabrix®-enhanced μCT were in similar range ~12–15%. In FTIRI analyses, corresponding biological variations were observed to be higher varying from 14–26% in the amide I region and 16–22% in the carbohydrate region throughout the depth of the tissue.

## Discussion

The results of this study suggest that PTA distribution mainly coincides with the collagen content in the superficial AC and relies on the balance dictated by the electric attraction forces of collagen and repulsion forces of the PGs within AC. The X-ray attenuation of PTA-labelled AC in CEμCT was observed to decrease substantially (14–40%) in the more superficial layers of the AC (tissue depth: 0–13%) after collagenase treatment and the distribution of PTA in AC corresponded to the Picrosirius red-stained histological slices by visual inspection. FTIRI showed more severe collagen degradation than the CEμCT with PTA suggested (difference of ~5–10% throughout the full tissue depth). However, the decrease in the amide I absorbance corresponded spatially to the decrease in the X-ray attenuation of PTA-labelled AC in CEμCT throughout the tissue depth. This confirms the hypothesis that the X-ray attenuation in the PTA-enhanced μCT of AC is associated with the collagen content. Chondroitinase ABC treatment did induce statistically significant changes of ~9–13% in the X-ray attenuation mainly in the middle and deep zones of the tissue (13–39% of tissue depth) in the chondroitinase ABC treated samples. Similar increase was observed in the Hexabrix®-labelled AC (Fig 3) after the chondroitinase ABC degradation. This correspondence to another negative contrast agent and the observed increase in the X-ray attenuation suggests that the PGs also have minor effect on PTA labeling, thus confirming the latter part of the hypothesis.

Histological, FTIRI, and uronic acid analyses revealed clearly that the GAG content was reduced also after the collagenase treatment. The structural analysis of PGs with agarose gel electrophoresis indicated that large PGs were markedly degraded by the collagenase. In human, there are 23 identified matrix metalloproteinases (MMPs), of which MMP-1, MMP-8, MMP-13 and MMP-18 are collagenases that cleave the interstitial collagen, but also a number of other extracellular matrix proteins [27]. The cleavage sites for collagenases have been identified previously for aggrecan [28] and link protein [29]. The bacterial collagenase from Chlostridium histolyticum used in this study has wide substrate degradation specificity [30], which suggests that it also is able to degrade bovine aggrecans, as supported by the agarose gel electrophoresis analysis. The degradation of aggrecan population decreases their average size so much that they can be expected to diffuse out of the tissue during the relatively long digestion times. Chondroitinase ABC degrades the chondroitin/dermatan sulfate, which itself diminishes the Safranin O staining of the tissue. However, the ability of the enzyme to degrade hyaluronan [31], liberating the aggrecan monomers, may have even bigger role for the loss of GAGs from the tissue. Almost a complete loss of Safranin O staining in the superficial and the middle zones of chondroitinase ABC-treated tissue suggests that keratan sulfate-containing aggrecan monomers are no longer present in the AC. The simultaneous loss of collagens and GAGs by the collagenase could be considered as the confounding factor for the minor, but clear decrease of the X-ray attenuation in PTA-stained AC detected with μCT.

Even though FTIRI, histology and uronic acid analyses clearly indicated presence of PG degradation, statistically significant changes in the X-ray attenuation of Hexabrix®-labelled AC after collagenase degradation was not observed. This suggests that high biological variation, determined from untreated samples, in CEμCT (CV of PTA: ~8–16%; and CV of Hexabrix®: ~12–15%) might affect the results by concealing the decrease or increase in X-ray attenuation. This variation could also affect detecting the changes in the X-ray attenuation of PTA-labelled AC in the deeper layers of the tissue, where the decrease in the attenuation is not statistically significant.

Regarding the suggested use of PTA labeling of AC in CEμCT for 3D histological evaluation of OA [4], the charactestics of PTA by revealing information on both PGs and collagen could be advantageous when determing the stage of OA *in vitro*. Especially if PG loss preceeds the changes of collagen matrix [2], the PTA-labelled AC in CEμCT could be used to determine changes not only on the integrity of the collagen matrix but also on the PG depletion.

In conclusion, the X-ray attenuation of PTA-labelled AC in CEμCT is associated mainly with the collagen content, whereas the PGs have minor effect on the X-ray attenuation of the PTA-labelled AC. PTA labeling of AC in CEμCT could provide a feasible tool for 3D histological evaluation of the OA.

## Supporting Information

S1 Table Data from depth-wise analyses and statistical tests. Means of the groups (n = 9), standard deviations, and relative changes between enzymatically treated groups and control group are shown in the table. Wilcoxon signed-rank test was used to determine if there were statistically significant difference between the groups. Statistical tests for each depth value was calculated separately and p-values less than 0.05 are bolded in the table.
(XLSX)
